# The Development of Cancer from Non-Malignant Diseases

**Published:** 1883-11

**Authors:** 


### The Development of Cancer from Non-Malignant Diseases.

Dr. Daniel Lewis read a paper on this subject before the Medical Society of the State of New York (*New York Med. Jour.*, June 30, 1883), which concludes with the following propositions:

1. Many diseases of non-malignant character are not only predisposing but exciting causes of cancer.
2. Such degeneration often occurs in patients who have no hereditary predisposition to cancer, and in those who are so predisposed the danger is imminent.
3. The recognition of the pre-cancerous stage of the disease is of the highest importance in its successful treatment.
4. While it is true that heredity is well attested in many cases, its importance has been greatly overestimated by all the older authorities and many writers of the present day.

